# Hospital Progress and Finance

**Published:** 1924-01

**Authors:** 


### HOSPITAL PROGRESS AND FINANCE.

### THE LISTER WARD—TO BE DEMOLISHED!

THE Board of the Glasgow Royal Infirmary has decided by 16 votes to 7 to demolish the historic Lister Ward. Mr. James Macfarlane was the chairman on what may yet be regarded as a notorious occasion. A third group of appeals for the retention of the ward arrived about the same time—from Montreal, Manitoba and Winnipeg. The protests have come from all over the world ; alternatives have been proposed, but the Board has chosen to disregard them and, in this matter, to betray posterity.

### THE PRINCE AT ISLINGTON.

THE Royal Northern Hospital was fortunate to have the Prince of Wales, who is its president, to open the new casualty department. This, Islington's War Memorial, has had £12,000 subscribed for it, and a further £4,000 is needed to pay the cost of the extension, which Mr. Percy Adams has designed. It already contains one endowed ward, thanks to the generosity of Mr. and Mrs. Martin Collins. The hospital needs money very badly. It had to close 70 beds in July, when the debt had increased to £50,000, and if it possessed 150 more beds it could fill them. Islington, indeed, claims to be the largest of the London boroughs with a population of 330,000, and the hospital's total number of beds is only 375. The form adopted for Islington's War Memorial is therefore very welcome, and the Prince's presence, to which, by the way, more than one generous donation from Welsh admirers was due, will help to emphasise the hospital's services to a population that it is exceedingly difficult to enlist collectively on its behalf. The task must be pursued, however, for the future of the hospital depends upon it.

### THE BOYS' GARDEN CITY.

THE new John Capel Hanbury Hospital at Woodford Bridge, the Boys' Garden city, is an interesting event in the history of Dr. Barnardo's Homes. The policy of the Council of that body has been to remove the children under its care to the country, and this has meant the creation of new institutions to replace those vacated in the City. The new hospital, which is named after its generous donor, replaces the institution in Stepney Causeway known as Her Majesty's Hospital, which was founded in 1889. The boys, it will be recalled, were moved last year to Goldings, near Hertford, and the new hospital is intended to serve, not only the Boys' Garden City, but all serious cases that can be moved from the Homes scattered about the country. It is interesting to add that the old Home in Stepney Causeway now accommodates only a few cripples, though these, too, would probably benefit by removal. Barnardo's Homes now provide for some 3,000 children, and their success has doubtless been due to the early age at which the children come under their care. The medical director of the Homes, Mr. Albert Carless, gave an interesting address at the opening of the new hospital. The move is another indication of the change that seeks to make town hospitals chiefly casualty and sorting stations on the road to treatment elsewhere.

### A STEP FORWARD AT READING.

THE contribution scheme connected with the Royal Berkshire Hospital which has been adopted by 106 parishes, and now numbers some 30,000 members, is being launched in Reading, where a similar amount of support is anticipated. It is remarkable that it should have spread last to the town, which is usually the originating centre, and the number of factories suggests that it will be easy to make a good start there. Some fears have been expressed that the large number of supporters is inadequately represented on the Committee of the hospital, but so far as we know the question of representation has not proved difficult elsewhere. Once the big firms join the scheme, large bodies of members will fall into their own groups, and can easily secure attention for any recommendations they may have to make. This surely is the decisive point, and the question whether it is accompanied by membership of the Hospital Committee is less important. The North has found that committees can be so unwieldy as to defeat their object ; bodies of subscribers can always make themselves heard should any of them have a real or fancied grievance.

### HOSPITAL ANNEXES.

THE reopening of the Catherine Gladstone Convalescent Home at Mitcham as an annexe of the London Hospital was, as we showed last month, made the occasion of insisting upon the value of these auxiliary institutions. They enable the mother hospital to devote its beds to diagnosis and immediate treatment and for the patient quickly to be sent away, but not home, to pursue recovery under better conditions of light and air. They are a partial remedy for the scarcity of beds which is the real weakness of the voluntary system, and one, oddly enough, that its critics rarely make the centre of their attack. At present, however, these annexes are the exception. But the day will come when every town hospital of any importance will be expected to possess them as a test of its efficiency.

### GREY OPERATING THEATRES.

IT is reported that the latest idea in American hospitals is to banish the colour white from operating theatres, ceilings, walls, and even the overalls of surgeons and nurses. From admitting that the colour on which the eyes of patients rest affects their spirits, the American enthusiast has gone on to assert that the eyes are especially fatigued by white. In substitution therefore, a light tan or grey is recommended for ceilings, walls, curtains, and furniture. Glazed white tiles are to be banished from the theatres, and grey enamel to be used for the equipment. From this the step to grey linen will probably follow, though not necessarily for sheets.

That white is tiring to the eyes is theoretically true. The question is whether it is sufficiently tiring to surgeons and patients to be forbidden altogether. Experience will show whether the new hospital in the American capital is an improvement or an experiment only.

### A NEW HOSPITAL FOR TORQUAY?

A SITE is under discussion for a new hospital at Torquay, and is complicated by the possible amalgamation in the future of Torquay and Paignton. If they join, a midway position for the new hospital would be desirable. But this is problematic, and the sites available for such a position are costly. The supporters of the Torbay Hospital therefore favour the offer of Major Kitson to sell to them the site known as Hengrave for £8,000, a sum which will provide fourteen acres, a big house, and several cottages. This district is expected to grow, and if the tramway could be extended, it would be generally accessible. The medical profession appears also to favour this solution, and if it is finally adopted an appeal for funds will quickly follow. It is now fourteen years since the Torbay Hospital was enlarged, and its future cannot remain undecided much longer.

### IMPROVED POOR LAW INSTITUTIONS.

THE Birmingham Board of Guardians is considering important proposals for extending the scope of the Poor Law hospitals under its control. The medical staffs at Dudley Road and Selly Oak are to be strengthened and increased in number, and an eye department and a dental department are contemplated at an early date. This will involve further accommodation for the medical and nursing staffs, at any rate at the Selly Oak Hospital. The plans have already been submitted to the Ministry of Health, and if the full quota of beds could be made available something would be done to relieve the hospital situation in Birmingham, which suffers from a scarcity of beds and, the voluntary hospitals at least, from serious want of money.

### THE AYR HOSPITAL'S NEW WING.

THE Ayr County Hospital has attained an enlarged status with the opening of its new wing raising the number of the hospital's beds to 107. The wing includes a children's ward with 14 beds, a men's ward with 10, 8 rooms for private patients, and overhead accommodation for twelve nurses. The building is regarded as fireproof, and has cost £14,000, which has been publicly subscribed. A distinguished company from different parts of Scotland attended the formal opening, and the vitality of the voluntary principle was emphasised. The addition of the rooms for private patients is important, for step by step the modern hospital is beginning to provide for all classes and to achieve the end which has already been gained in America for all sections of the population. Until this has been accomplished a hospital service will remain incomplete, though it is difficult to expand in this direction until the number of beds is more adequate to the general need throughout the country.

### TWENTY YEARS AFTER.

THE long delay in beginning the Carter Bequest Hospital is apparently over, and Middlesbrough will shortly possess an institution with fifty-two beds on the three to four acres of land secured in Cambridge-road, Linthorpe. A tender has been accepted for £22,500 and the specifications include small wards for patients of moderate means. It was in 1904 that Mr. Carter left £40,000 to build a hospital. The estate was chiefly cottage property; there were mortgages to be paid off, and the interval of twenty years has enabled the trustees to do this, and to devote £25,000 to building and equipping the institution. The delay has provided not only a new hospital but one with a small endowment round which voluntary support should readily grow out of gratitude to the far-sightedness of the founder.

### A CORNISH COTTAGE HOSPITAL.

SINCE the Helston Cottage Hospital was opened last year, thirty-nine patients have been admitted, eleven of whom have entered the private wards. The charges are three guineas a week for residents and four guineas a week for strangers or visitors. This record shows that the hospital is not only in request for minor ailments, but that private patients rely upon its services. The value of cottage hospitals has been lately challenged here, though in America a movement of envy has led to the demand for their creation in that country. The experience of Helston leads one to inquire why new cottage hospitals in other places cannot perform a similar service, and thus help to relieve congestion elsewhere.

### GUY'S CHRISTMAS CARDS.

AMONG the institutions which issue Christmas cards each year is Guy's, which, thanks to Messrs. Ash, has received a new series, to be sold for the hospital's benefit. The series this time has no pictures or illustrations, but possesses the hospital crest stamped in colours, and a bow of the hospital ribbon at the side. People of middle age can remember the public competition for words and designs for Christmas cards in which many Academicians took part. Tennyson, of course, was invited to contribute, but declined despite the large fee that he was offered by the firm that arranged the competition. If the most were to be made of the idea by hospitals, a collection of those issued by all of them would be desirable, for there is no reason why the hospital Christmas card should not be as remarkable in its own kind as, for example, the Medici Society's cards with their reproductions of old pictures.

### MARRIED QUARTERS FOR MEDICAL OFFICERS

The Visiting Committee of the Brighton Mental Hospital have recommended the Town Council to provide married quarters for the first Assistant Medical Officer, and the house occupied by the late holder of this office, who is now the Medical Superintendent, will probably be purchased for the purpose. The old grievance that no one in the mental hospital service except the Medical Superintendent could marry is being slowly removed.

